# Genomic characterisation of an extended-spectrum β-Lactamase-producing *Klebsiella pneumoniae* isolate assigned to a novel sequence type (6914)

**DOI:** 10.1186/s13099-024-00662-4

**Published:** 2024-11-15

**Authors:** Muiz O. Akinyemi, Oluwawapelumi A. Oyedele, Mariska S. Kleyn, Bukola A. Onarinde, Rasheed A. Adeleke, Chibundu N. Ezekiel

**Affiliations:** 1 Leeds Institute of Health Sciences, University of Leeds, Leeds, UK; 2 Department of Microbiology, Babcock University, Ilishan Remo, Ogun State Nigeria; 3 Unit for Environmental Sciences and Management, North-West University (Potchefstroom Campus), Potchefstroom, South Africa; 4 Institute of Bioanalytics and Agro-Metabolomics, Department of Agrobiotechnology (IFA-Tulln), University of Natural Resources and Life Sciences, Vienna (BOKU), Konrad Lorenz Str. 20, Tulln, 3430 Austria; 5 National Centre for Food Manufacturing, University of Lincoln, Holbeach, PE12 7PT UK

**Keywords:** Animal milk, Draft genome, *Klebsiella pneumoniae*, Multidrug resistance, Nigeria

## Abstract

**Background:**

Cow milk, which is sometimes consumed raw, hosts a plethora of microorganisms, some of which are beneficial, while others raise food safety concerns. In this study, the draft genome of an extended-spectrum β-lactamase-producing *Klebsiella pneumoniae* subsp. *pneumoniae* strain Cow102, isolated from raw cow milk used to produce traditional foods in Nigeria, is reported.

**Result:**

The genome has a total length of 5,359,907 bp, with 70 contigs and a GC content of 57.35%. A total of 5,244 protein coding sequences were detected with 31% mapped to a subsystem, and genes coding for amino acids and derivatives being the most prevalent. Multilocus sequence typing revealed that the strain had new allelic profile assigned to the novel 6914 sequence type possessing capsular and lipopolysaccharide antigen K locus 122 with an unknown K type (KL122) and O locus O1/O2v2 with type O2afg, respectively. A total of 28 resistance-related genes, 98 virulence-related genes, two plasmids and five phages were identified in the genome. The resistance genes *oqxA*, *oqxB* and an IS*3* belonging to cluster 204 were traced to bacteriophage *Escher 500,465*. Comparative analysis predicted one strain specific orthologous group comprising three genes.

**Conclusion:**

This report of a novel sequence type (ST6914) in *K. pneumoniae* presents a new allelic profile, indicating ongoing evolution and diversification within the species. Its uniqueness suggests it may represent a locally evolved lineage, although further sampling would be necessary to confirm this hypothesis. The strain’s multidrug resistance, virulence gene repertoire, and isolation from animal milk render it a potentially significant public health concern, underscoring the importance of genomic surveillance in non-clinical settings to detect emerging strains. Further research is required to fully characterise the capsular K type of ST6914.

**Supplementary Information:**

The online version contains supplementary material available at 10.1186/s13099-024-00662-4.

## Background

In recent years, there has been growing concern about the emergence and spread of antibiotic resistance in bacteria [1]. One of such examples involve the rising incidence of various species, especially strains of *Klebsiella pneumoniae*, acquiring the capability to synthesize extended-spectrum β-Lactamase (ESBL) enzymes that confer resistance to a broad range of β-lactam antibiotics [2]. β-Lactam antibiotics act by interfering with bacterial cell wall formation, binding specifically and covalently to essential inner-membrane bound enzymes known as penicillin-binding proteins (PBPs) [3]. These PBPs are vital for the last steps of peptidoglycan molecule cross-linking, a critical component of the rigid network that makes up a bacterial cell walls [3]. This mechanism of action is effective against both Gram-negative and Gram-positive bacteria, making β-Lactam antibiotics broad-spectrum antimicrobial agents [3]. To defend themselves against β-lactam antibiotics, such as penicillins, cephalosporins, monobactams, and carbapenems, which are widely used to treat bacterial infections, these bacteria, especially members of the Enterobacteriaceae family have evolved ESBLs as a protective mechanism [3–5]. These enzymes inactivate β-lactam antibiotics by hydrolysing their β-lactam ring, transforming them into compounds that lack affinity for PBPs and, thus, cannot disrupt bacterial cell wall stability [3–5]. By neutralizing the beta-lactam antibiotic’s action, ESBLs grant bacteria a highly effective form of resistance, creating a serious problem in the treatment of infection by ESBL-producing bacteria.

Members of the *Klebsiella* genus, a Gram-negative non-motile, encapsulated, facultative anaerobic bacterium, within the family Enterobacteriaceae cause a wide range of infections in both humans and animals. *Klebsiella pneumoniae* is a versatile bacterium that can be found in various environments, including the oral cavity, skin, and gastrointestinal tracts of different mammals [6]. While normally present as a part of the human microbiota, this bacterium can also act as an opportunistic pathogen, leading to a range of infections such as pneumonia, septicaemia, urinary tract infections, and soft tissue infections, among others [6].

In recent years, the World Health Organization (WHO) has recognized *K. pneumoniae* as being among the top priority pathogens due to the significant morbidity and mortality rates associated with its infections and status as a major worldwide reservoir and vector for antibiotic resistance [1, 7]. As a result, there is a growing demand for genotyping this important pathogen to better understand its genetic and patterns of strain-specific transmission both within and between species [8]. One of the molecular methods commonly employed for this purpose is multilocus sequence typing (MLST) based on housekeeping genes, allowing the characterization of the genetic relationship between different bacterial strains. *Klebsiella* strains are largely oligoclonal [9], which means they can be assigned into several unique sequence types (STs). This method of classification provides unambiguous and reliable data, which allows researchers and healthcare professionals to gain valuable insights into the genetic diversity and transmission patterns of this pathogen.

In Nigeria, a significant proportion of the available data that employed molecular techniques to identify *Klebsiella* strain is predominantly derived from studies conducted in healthcare facilities and medical settings [10, 11]. Studying the genomic characteristics of ESBL-producing *K. pneumoniae* isolated from alternative sources, such as animal milk, could offer fresh perspectives on the transmission of these opportunistic pathogens across different niches in Nigeria. Moreover, genome typing of *Klebsiella* strains from various sources will provide resources for bio-surveillance. This research also contributes to understanding antibiotic resistance dissemination as well as the development of targeted interventions and preventive measures to mitigate the spread of *K. pneumoniae* in both animal and human populations.

## Results and discussion

### Antimicrobial susceptibility profile

*Klebsiella* species are major global reservoirs and conduit of drug resistance genes between various bacterial niches [12]. We report the draft genome sequence of an ESBL-producing *K. pneumoniae* subsp. *pneumoniae* strain Cow102 previously isolated from cow milk [13, 14]. The phenotypic antimicrobial susceptibility profile of strain cow102 showed resistance to cephalosporins, carbapenem, and drugs from different classes of antibiotics including aminoglycosides (streptomycin), quinolone (ciprofloxacin, nalidixic acid), beta-lactams (amoxicillin + clavulanic acid, ampicillin, cefotaxime, ceftazidime, piperacillin), folic acid synthesis inhibitor (trimethoprim/sulfamethoxazole), monobactams (aztreonam), fosfomycin, and tetracycline. The strain was identified as an ESBL producer due to its resistance to aztreonam, cefotaxime, ceftazidime, and ceftriaxone, which are commonly used as indicators for ESBL production. The antimicrobial resistance profile and ESBL-producing status of strain Cow102 is consistent with characteristics commonly observed in ESBL-producing *K. pneumoniae* isolates from clinical, environmental, and animal sources in Nigeria [11, 15–18]. This suggests the pervasive nature of antibiotic resistant *K. pneumoniae* across different ecological niches in the country.

### Haemolysis test

Strain cow102 was non-hemolytic on blood agar and lacked the genes hlyA and cnf-1 that code for this function. This finding contrasts with some clinical isolates reported in various states in Nigeria [11, 15, 16, 19], suggesting potential differences in virulence mechanisms between clinical and environmental strains.

### General genome features

**Fig. 1:**
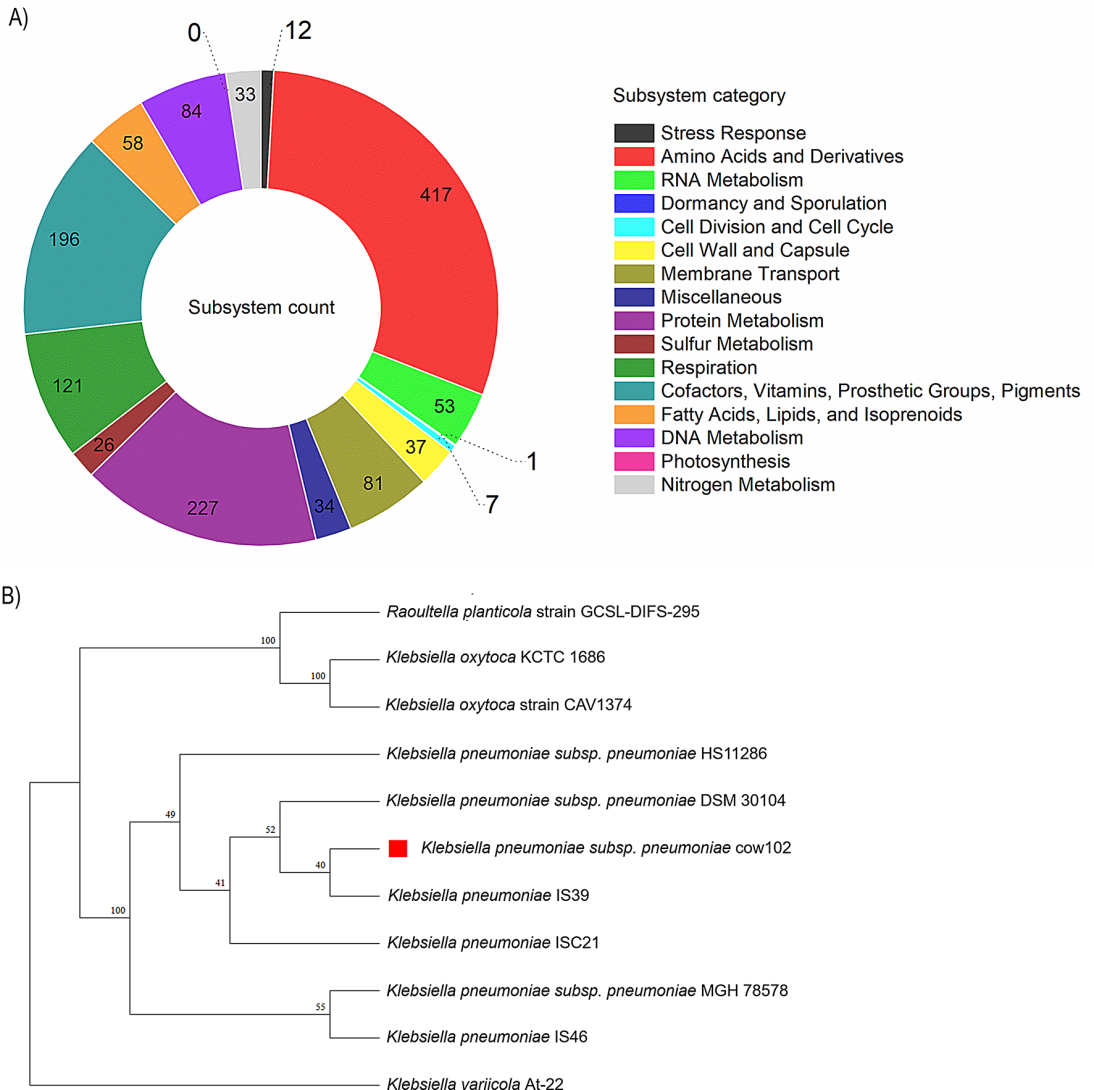
Subsystem distribution and phylogenetic analysis. **A**. Overview of the subsystems for *Klebsiella pneumoniae* subsp. *pneumoniae* strain Cow102 **B.** Whole genome phylogenetic tree of *K. pneumoniae*

Illumina sequencing of the genomic DNA of strain cow102 generated 12,005,232 total read pairs (2 × 150 bp) giving approximately 336 × coverage. The assembly genome totalled 5,359,907 bp with 70 contigs, an N50 value of 376,296 bp, and a GC content of 57.35%. The assembly was characterized by CheckM as 100% complete with 0.1% contamination and had coarse and fine consistencies of 99.4% and 97.8%, respectively. Genome annotation performed using RASTk identified a total of 5,244 protein coding sequences (CDS), 72 transfer RNA (tRNA) genes, and 5 ribosomal RNA (rRNA) genes. There were 4,605 proteins with functional assignments and 639 hypothetical proteins in the annotation. Among the proteins with functional assignments, 1,457 possessed Enzyme Commission (EC) numbers, 1,208 had Gene Ontology (GO) assignments, and 1,065 contained KEGG pathway mappings. Also, about 31% of the CDS was mapped to a subsystem. Genes responsible for amino acids and derivatives (417 ORFs), protein metabolism (227 ORFs), and cofactors, vitamins, prosthetic groups, and pigments (196 ORFs) were abundant among the SEED subsystem categories [20]. An overview of the subsystem categories and feature counts is shown in Fig. 1A while the genome annotation can be viewed on Figshare [21].

### Phylogenetic analysis, Multilocus sequence typing (MLST) and capsular typing

The whole-genome phylogenetic analysis showed that strain Cow102 is a sister taxon to *K. pneumoniae* subsp. *pneumoniae* strain IS39 and a close relative to ISC21, DSM30104, and HS11286 all of which are within the same monophyletic group (Fig. 1B). Monophyletic groups, also known as clades, are groups of organisms that belong to the same taxon and share a most recent common ancestor. Similar to Cow102, all related strains were reported as multidrug-resistant β-lactamase producers. Strains IS39 and ISC21 were recovered in Austria from hospital environments [22], DSM30104 was isolated in Republic of Korea from the blood of a septicaemia patient [23], and HS11286 was recovered in China from human sputum [24]. Multilocus Sequence typing analysis revealed that the current isolate had a new allelic profile [(*gapA* (2), *infB* (3), *mdh* (70) *pgi* (1) *phoE* (275), *rpoB* (44) and *tonB* (39)], assigned as sequence type 6914 based on the standard 7-gene MLST scheme, initially defined by Diancourt et al. [25]. eBURST analyses showed that ST6914 clustered closely with ST494, ST4138, ST4139 and ST6476 (Figure S1A). The current strain, ST6914 is a triple-locus variant to all closely clustered ST, differing at the *phoE*,* rpoB* and *tonB* loci. When compared with phylogenetically related strains, Cow102 shared only one or two loci with IS39 and ISC21 strains both unassigned STs, as well as with DSM30104 and HS11286, which belong to STs 3 and 11. The serotyping of ST6914 revealed a 99.96% match with K locus 122 with an unknown K type (KL122) for the capsular (K typing) antigen (Figure S1B) using the wzi sequencing K-typing method [26], and a 98.51% match with O locus O1/O2v2 with type O2afg for the lipopolysaccharide (O typing) antigen (Figure S1C).

### Antimicrobial resistance genes

**Fig. 2:**
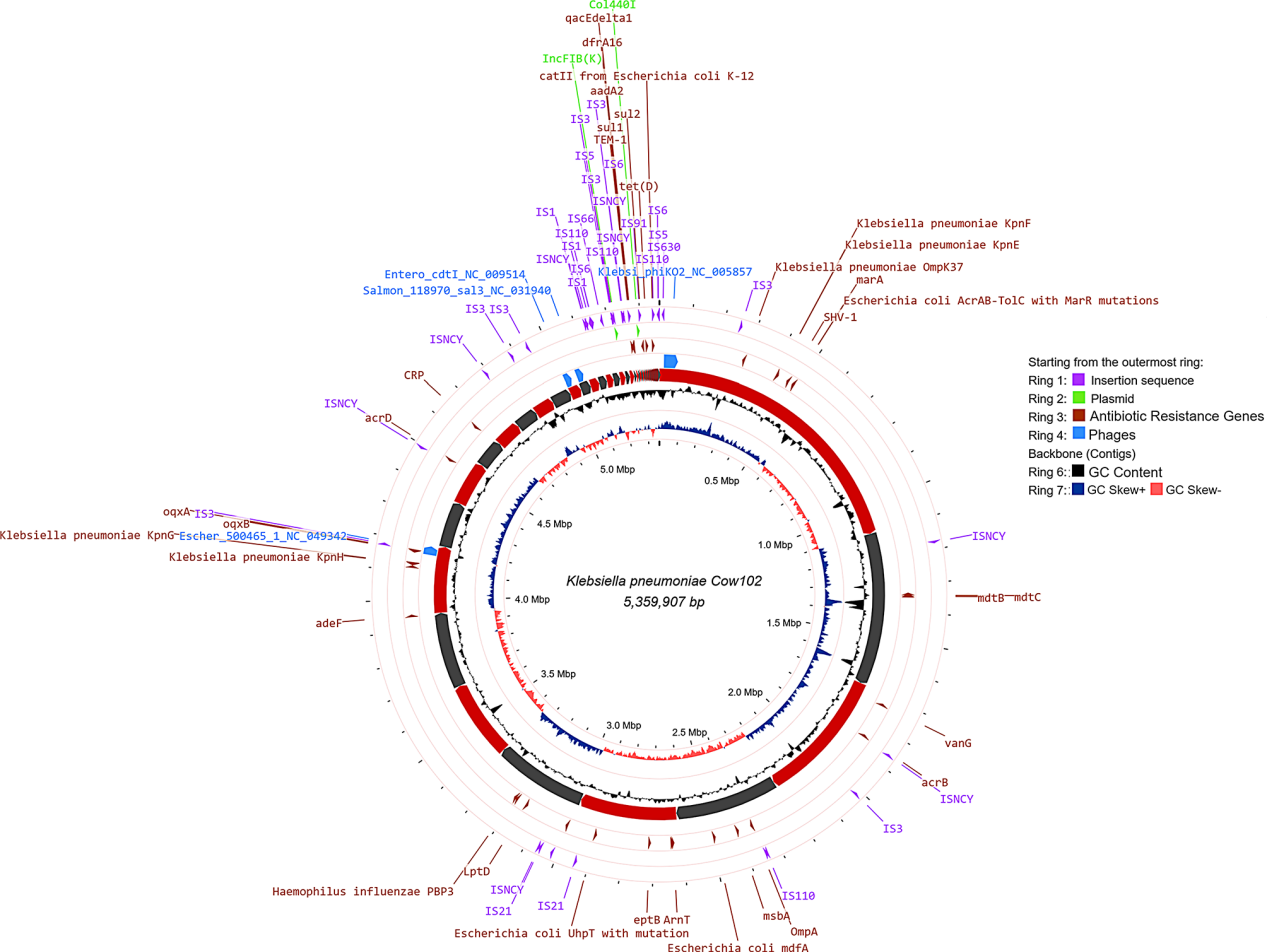
Circular genomic map of *Klebsiella pneumoniae* subsp. *pneumoniae* strain Cow102 showing starting from the outermost ring: ring 1: insertion sequence ring 2: plasmids (coloured green), ring 3: antibiotic resistance genes (coloured red), ring 4: phage (coloured blue), ring 5: contigs from strain Cow102 (coloured red and black), ring 6: GC content (coloured black), ring 7: GC skew (coloured red and blue)

Several studies in Nigeria reported drug resistant *Klebsiella* strains in raw animal milk and milk products; however, genomic data and serotypes of the reported strains are rare [18, 27]. Molecular characterization of the resistance factors associated with strain Cow102 revealed the presence of 38 Antibiotic Resistance Genes (ARGs) (Table 1; Fig. 2), out of which 24 genes conformed with the phenotypically derived resistance profile. The resistance genes identified in strain Cow102 include core chromosomal resistance genes belonging to the *bla*_SHV−1_ family, which confers resistance to beta-lactam antibiotics. In addition, acquired genes such as *aadA2*, an aminoglycoside nucleotidyltransferase gene; *catA2* and *catII*, which confer resistance by enzymatic inactivation of amphenicol antibiotics; *TEM* families, which confer resistance through similar mechanisms as *bla*_SHV−1_ family; and *Escherichia coli fosA* and *oqxA* and *oqxB* gene conferring resistance to fosfomycin and quinolones, respectively, were found. Furthermore, the presence of the efflux pump gene, *tet (D)*, which provides resistance to tetracycline, was expected (Table 1; Fig. 2). This is likely due to the common practice among veterinary and para-veterinary professionals in Nigeria to prescribe oxytetracycline for treating diseases [28, 29].

**Table 1:** Antimicrobial resistance genes identified in *Klebsiella pneumoniae* subsp. *pneumoniae* strain Cow102, conferring resistance to antibiotics tested using the disk diffusion method

Resistance gene	Antibiotic Class	Antibiotic (phenotype observed)
*aadA2b*	aminoglycoside	Streptomycin (resistant)
*bla*_SHV−1_a	beta-lactam	Amoxicillin (resistant) ^a, b,c, d,f, g,n^
*bla*_SHV−11_^b^		Amoxicillin + clavulanic acid (resistant)^d^
*bla*_SHV−13_^c^		Ampicillin(resistant) ^a, b,c, d,f, g,n^
*bla*_SHV−26_^d^		Ampicillin + clavulanic acid (resistant)^d^
*bla*_SHV−70_^e^		Aztreonam (resistant)^c, e,g^
bla_SHV−78_^f^		Cefotaxime (resistant) ^c, e,g^
*bla*_SHV−98_^g^		Ceftazidime (resistant) ^c, e,g^
*bla*_SHV−145_^h^		Ceftriaxone (resistant) ^c, e,g^
*bla*_SHV−161_^i^		Piperacillin (resistant) ^a, b,c, d,f, g,n^
*bla*_SHV−179_^j^		
*bla*_SHV−185_^k^		
*bla*_SHV−194_^l^		
*bla*_SHV−199_^m^		
*bla*_TEM−1B_^n^		
*catA2*	amphenicol	Chloramphenicol (intermediate)
*dfrA16* ^*q*^	folic acid synthesis inhibitor	trimethoprim^o, q^/sulfamethoxazole^p^ (resistant)
*fosA*	fosfomycin	Fosfomycin (intermediate)
*fosA5*		
*fosA6*		
*oqxA* ^*o*^	Quinolone and folic acid synthesis inhibitor	Ciprofloxacin (intermediate)
*oqxB* ^*o*^		Nalidixic acid (resistant)
*sul1* ^*p*^	folic acid synthesis inhibitor	
*sul2* ^*p*^		
*tet(D)*	tetracycline	Tetracyclin (resistant)

Observed phenotypes are listed in parentheses next to each antibiotic in the corresponding column

Key: Superscripts represent combination of resistance gene conferring resistance to an antibiotic

The genome as shown in Fig. 2 includes several additional genes associated with antimicrobial resistance that were not phenotypically tested, and their presence alone does not confirm resistance. These include *katG* gene and operons *marA*,* marB*, and *marR*, which are involved in enzymatic catalysis of antibiotics. The *BcrC* gene serves as a protective protein for antibiotic targets.The *gdpD* and *pgsA* genes are involved in altering the charge of the cell wall, while *occD6/OprQ* and *oprB* modulate antibiotic permeability. The presence of *acrAB-tolC*, *emrAB-tolC*, *H-NS*, and *oxyR* suggests the role of regulatory factors in modulating the expression of antibiotic resistance genes. Additionally, the genome harbours other ARGs that were acquired through horizontal gene transfer such as the *E. coli uhpT* gene, which carries a mutation conferring resistance to fosfomycin, *mdfA* gene, an efflux pump, and multidrug transporter, as well as the *Haemophilus influenzae PBP3* gene which confers resistance to beta-lactam antibiotics (Fig. 2). Comprehensive details of ARGs, their orientation and genomic location is presented in Supplementary data S1.

### Virulence factors

Pathogens establish infections and achieve long-term survival within their host by deploying an array of virulence factors. These factors, often manifested as complex cellular structures or specialized strategies which facilitate critical functions such as host colonization, immune evasion, nutrient acquisition, and responsiveness to environmental cues [30–32]. However, it is important to note that the relationship between sequence type and virulence characteristics in *K. pneumoniae* is complex and not always straightforward. While certain sequence types may be associated with increased virulence, the presence of specific virulence genes does not necessarily correlate directly with the assigned sequence type [9, 33]. The pathogenicity of a *K. pneumoniae* strain is influenced by multiple factors beyond just its sequence type, including the specific combination of virulence genes, their expression levels, and host-pathogen interactions [9]. Profiling of virulence features in the current strain revealed the presence of 98 virulence factors (Supplementary Table 1) classified into 11 categories. These categories are: (i) adherence (15 genes) which allow attachment to host cells, (ii) antimicrobial activity/competitive advantage (3 genes), (iii) biofilm formation (10 genes), this is defined by the ability of microbial cells to aggregate and form a community encased in self-produced polymetric matrix [31], (iv) effector delivery system (25 genes), these are elements that facilitate the delivery of secreted effector proteins into host cells, these effector proteins alter or interfere with host processes allowing the bacteria invade, adhere, multiple and sometimes persist in the host [31], (v) exotoxins (2 genes) are secondary metabolites secreted by a microbe which interrupts or dysregulates cellular functions of the host [32], (vi) immune modulation (16 genes) allows the suppression or evasion of host immune system [30], (vii) invasion (2 genes), (viii) motility (3 genes), (ix) nutritional/metabolic factors (14 genes), (x) regulation (7 genes), and xi) other (1 gene). Hypervirulence in *Klebsiella* is mainly characterised by factors such as capsule type, siderophores production, lipopolysaccharide and fimbriae [34, 35]. As a result, some studies on *Klebsiella* focus on related genes such as *mrkD*,* entB*,* iutA*,* rmpA*,* ybtS*,* ycfM*,* kfu*, and *allS* [34–36]. Cow102 has an unclassified capsule serotype which indicates its distinction from the known serotypes of classical *K. pneumoniae* (K1 – K79), hypervirulent *K. pneumoniae (*K1, K2, K5, K16, K20, K54, K57, KN1) and multidrug resistant hypervirulent *K. pneumoniae (*K1, K2, K16, K20, K54, K62, K64 and K47) [34]. The genome of strain Cow102 contains multiple genes linked to virulence in *K. pneumoniae*, including *mrkD*, an adhesion factor that encodes the type 3 fimbrial adhesin, facilitating binding to the extracellular matrix. Additionally, siderophore systems such as *entB*, which is involved in synthesizing enterobactin (a catecholate siderophore), and *iutA*, encoding the receptor for aerobactin (a hydroxamate siderophore), are present in the genome (Supplementary Table 1). These virulence factors contribute to the strain’s ability to acquire iron (siderophores) and adhere to host surfaces (fimbrial adhesin) [34]. However, some genes commonly associated with hypervirulence in *K. pneumoniae*, such as *rmpA*,* ybtS*,* ycfM*,* kfu*, and *allS* [35], were not found in Cow102.

### Mobile genetic elements

Mobile genetic elements (MGEs) are enzymes or other protein encoding non-chromosomal DNA that facilitate the relocation of DNA segments either within the genome or between microorganisms [37]. Examples of MGEs include transposons (e.g., integrons, insertion sequences, gene cassettes), plasmids and phages. Figure 2 provides a visual illustration of where insertion sequences, plasmids bacteriophage are located within the CDS of the Cow102 strain. A total of 23 complete and 12 partial insertion sequences (IS) classified into ten IS families: IS*1*, IS*3*, IS*5*, IS*6*, IS*21*, IS*110*, IS*66*, IS*91*, IS*630*, and IS*NCY* in the genome of strain Cow102 (Fig. 2). A comprehensive list of all identified IS, along with their corresponding CDS positions and terminal inverted repeats (TIR) is presented in Supplementary data S2. The only plasmids found in the present genome were IncFIB(K) and Col440I. These plasmids were respectively detected in 16 (67%) and 11 (46%) of 24 *K. pneumoniae* isolates from powdered milk from Germany [38]. Studies have shown that the conjugative IncFIB plasmid group, known for their role in the distribution of antibiotic resistance and virulence genes, are commonly found in various strains of *K. pneumoniae*, contributing to their multidrug-resistant profiles [24, 39–43]. One such study includes a report from Brazil, where 25 out of 27 carbapenem-resistant *K. pneumoniae* isolates from a hospital environment were found to carry variants of the IncFIB plasmid [42]. In our study, we also observed that this plasmid was present in all the strains from Nigeria which we retrieved from the NCBI database. (Supplementary Data 3). IncFIB plasmids in *Klebsiella* species are crucial vectors for the transmission of ARGs and virulence genes, contributing to the pathogenicity and antimicrobial resistance of these bacteria [42, 44]. This highlights the need for further research on the genomic features and evolutionary dynamics of variants of IncFIB plasmids to combat the spread of resistance and virulence traits. Additionally, five bacteriophages were detected in Cow102, of which three (*Klebsi phiKO2*,* Escher 500465*,* Entero cdtI)* were intact, one (*Salmon 118970*) was incomplete, and one (*Escher RCS47*) yielded inconclusive results. The nucleotide sequence of all phagesis publicly available on Figshare. The ARGs *oqxA*, *oqxB* and an IS*3* belonging to cluster 204 were traced to bacteriophage *Escher 500,465*. While this phage has been documented in the genomes of *Klebsiella* strains [36] and other enterobacteria like *Pseudomonas* [45] and *Salmonella* [46], this appears to be the first instance where it has been linked to the *oqxA* and *oqxB* genes in *Klebsiella*. *Escher 500,465* has previously demonstrated the capacity to carry ARGs, as evidenced by its harbouring of *bacA* and *tet(M)* genes in *Salmonella* [46]. This finding expands our understanding of how antibiotic resistance genes can be transmitted among bacterial populations through phage-mediated gene transfer.

### Comparative genomics

**Fig. 3:**
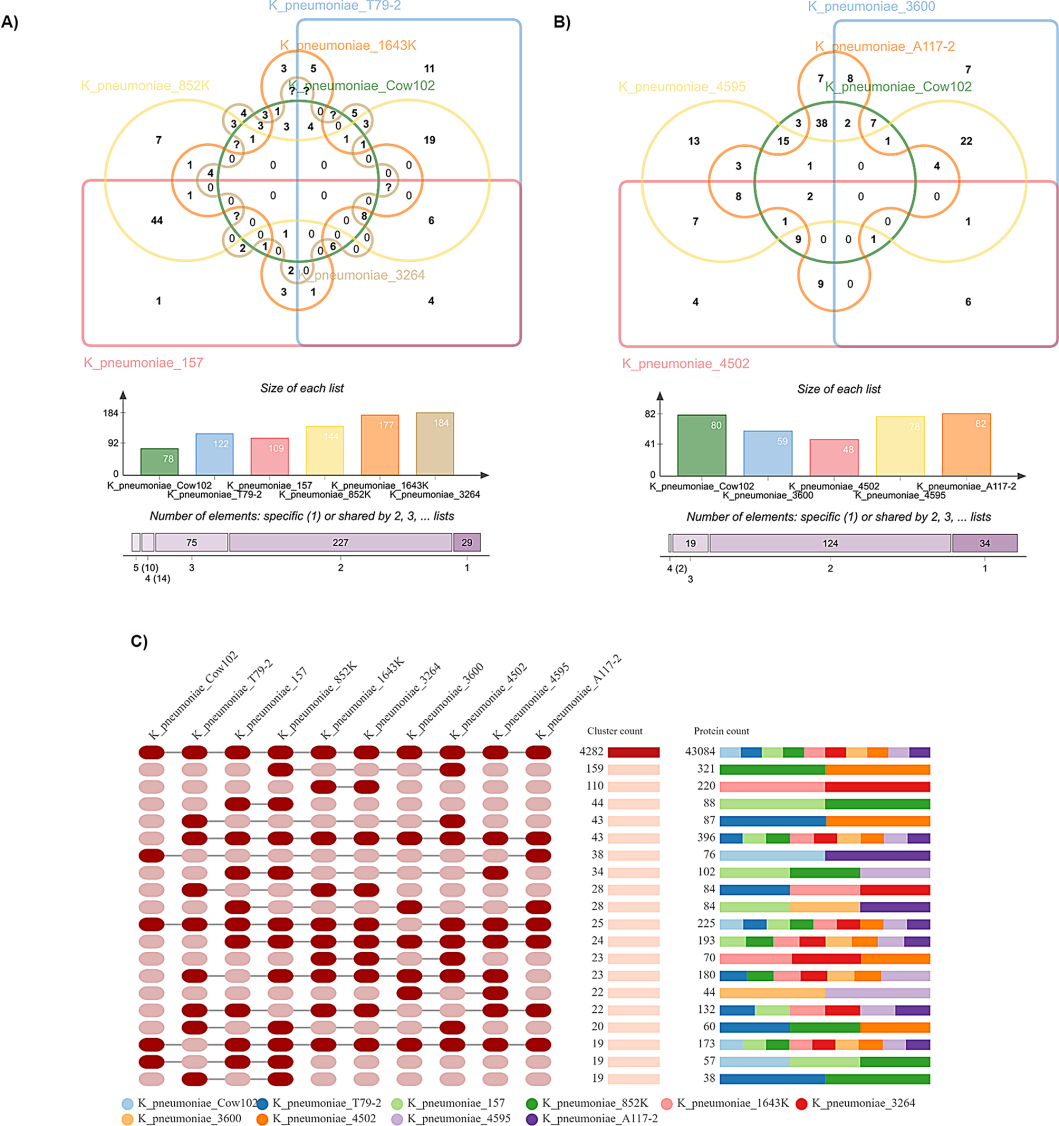
Comparative genome analysis represented as (**A**, **B**) Venn diagram illustrating the distribution of shared and specific clusters of orthologous groups between *Klebsiella pneumoniae* subsp. *pneumoniae* strain Cow102 and nine type strains of *K. pneumoniae* reported in Nigeria. And **C**) occurrence table showing groups of gene clusters, including the cluster count (the number of gene clusters shared between species) and the protein count (the number of protein members in the shared clusters across these strains). Each row represents an orthologous gene cluster spanning multiple species, while each column corresponds to a different closely related strain

A comparative analysis was conducted between the genome of the current strain and nine other strains of *K. pneumoniae* reported in Nigeria, obtained from NCBI database. A first glance at the genomic comparison analysis suggested a high level of sequence similarity with pairwise average nucleotide identity (ANI) ranging from 99.1 to 99.9% (Figure S2). In addition, pairwise genome alignments showed high collinearity among all strains with sequence alignment percentages of about 85% and 90% (Figure S2). A closer look at the variations between the genomes using DNAdiff 1-to-1 alignment blocks found significant differences (*p* < 0.05) in sequence inversions, insertions/deletions (indels), relocations and translocations between the assemblies and the examined strains (Supplementary data S3). The 1-to-1 alignment blocks are specific DNA regions that can be directly and uniquely matched between two genomes or sequences being comparison. These blocks represent segments where there is a one-to-one correspondence, meaning each sequence in a reference genome has exactly one matching counterpart in the query genome, without duplications or significant rearrangements [47]. The most common structural rearrangements discovered were translocations (x̄=105), which occurred between neighbouring 1-to-1 alignment blocks in the mapping of the reference (Cow102) and query sequence (nine strains obtained from NCBI). Additionally, there was an average of approximately nine breaks in the alignment where adjacent 1-to-1 alignment blocks were in the same sequence (relocations), and two somewhat unusual breaks where adjacent 1-to-1 alignment blocks were inverted with regard to each other (inversions). Furthermore, an average of 221 insertion events, three tandem duplication insertion events (TandemIns), 32,995 Single Nucleotide Polymorphism (SNPs) and 2629 Single Nucleotide. The similarity between *K. pneumoniae* strains extends to their entire proteomes. Among the ten strains compared, a total of 1789 protein coding genes were predicted (Supplementary data 4). Of these, 1617 genes (90.4%) were assigned orthologous groups (OGs), while 172 genes (9.6%) remained unassigned. Orthologous genes or orthologs are groups of genes shared by different species or strains evolved from a common ancestry gene but diverged from each other because of a speciation event [49]. OrthoVenn analysis of orthologous cluster structure identified 6,299 clusters, 53,361 proteins, and 2,098 singletons (Fig. 3). Figure 3A and B display the orthologous groups in each strain, highlighting both unique and shared homologous gene clusters among them. Of these, 4,282 clusters and 43,084 proteins were shared across all strains (Fig. 3C). The ortholog analysis conducted on Cow102 revealed the presence of 4796 COGs and 5037 proteins, including 196 singletons. Additionally, three proteins were found to be unique to the strain. These unique proteins were involved in carbohydrate metabolic processes (GO:0005975), specifically phospho-cellobiase and 5-oxoprolinase subunit A3, as well as fatty acid biosynthesis (GO:0006633) through biotin-dependent acyl-coenzyme A carboxylase alpha3 subunit.

Results of comparison of the nine strains recovered from NCBI database with Cow102 revealed that 10 proteins were shared with strain T79-2, isolated from chicken faeces; 38, 9, and 1 proteins were shared with strains 157, 4502, and A117-2, respectively, isolated from blood or wounds; and 16, 15, 7, 3, and 1 proteins were shared with strains 852 K, 4595, 3600, 3264, and 1643 K, respectively, all isolated from human urine (Fig. 3). The functional enrichment analysis conducted on the shared OGs between Cow102 and the nine strains revealed the presence of strain to strain specific proteins with molecular functions. Some of these proteins that could play a role in the development or distribution of drug resistance genes include the excisionase protein (GO:0032359) with the gene ontology term “provirus excision,” shared with T79-2. This protein facilitates the excision of prophage from the host genome through site-specific recombination. Additionally, a recombination-associated protein RdgC (GO:0006310) was shared with strain 157, and a family 20 transposase (GO:0006313) was shared with strains T79-2, 4502, 852 K, and 4502. This protein is required for the transposition of an insertion element. The CRISPR system Cascade subunit CasA (GO:0043571 and GO:0051607), an adaptive immune system that provides protection against mobile genetic elements, was shared with 4502. Also present among the strain-to-strain specific proteins detected were those involved in biological processes such as the heavy metal binding protein N-acetylcysteine deacetylase (GO:0046872) shared with 4502 and 852 K. Additionally, the S-(hydroxymethyl) glutathione dehydrogenase protein (GO:0046294) involved in formaldehyde catabolic process and the S-formylglutathione hydrolase FrmB (GO:0006069) involved in ethanol oxidation were shared with strain 3600. the analysis revealed a 2,3-diketo-L-gulonate reductase (GO:0070403) protein, an NAD + binding protein, which is shared with strain 4595.

Strain 852 K had the highest number of plasmids among all the strains analysed, with a total of seven (Supplementary data S3) while strains T79-2 and 4595 were found to contain four plasmids, placing them among the top in this aspect. Only strain A117-2 shared the same number and type of plasmid, Col440II and IncFIB(K), with Cows102.

## Conclusion

*Klebsiella pneumoniae subsp. pneumoniae* cow102 an ESBL-producing, pathogenic strain was isolated from raw cow milk. The genomic characterization of strain Cow102 reveals a novel sequence type (ST6914) with a unique capsular and lipopolysaccharide antigen profile. This information is crucial for enhancing the surveillance of *K. pneumoniae* strains, as it allows for the identification and tracking of specific clones that may be associated with resistance profiles or virulence factors. Surveillance systems can use this data to monitor the emergence and spread of MDR strains and to inform public health interventions. We investigated antimicrobial resistance genes and virulence factors related to pathogenicity *in K. pneumoniae subsp. pneumoniae* Cow102, this revealed the presence of multiple genes such as the *bla*_SHV_ family encoding resistance to wide range of antibiotics, including cephalosporins, carbapenems, aminoglycosides, quinolones, and tetracycline. This indicates a high level of multidrug resistance, which poses a challenge to the effective treatment of potential infections caused by this strain. Clinicians and veterinarians may need to consider alternative or combination therapies, possibly involving newer antibiotics or synergistic combinations to treat such infection. The identification of specific resistance genes, such as those belonging to the *bla*_SHV_ family and other acquired genes like *aadA2*,* catA2*, and *catII*, can guide antimicrobial stewardship programs. A comparative analysis of the genome of this strain and those of T79-2, 157, 4502, A117-2, 852 K, 4595, 3600, 3264, and 1643 K revealed significant homology in terms of the genomic structure, gene content and virulence factors with average nucleotide identity ranging between 99.1 and 99.9%. Despite the similarity in genome sequences and content, there were differences in functional proteins and number of plasmids. Cow102 harbours three unique proteins when compared to the other strains, all three proteins serve biological functions. The IncFIB(K) plasmid was detected in all the strains although the copy present in Cow102 was not found to carry antibiotic resistance genes.

The findings from this study can inform more effective treatment strategies, enhance bio-surveillance efforts, and guide public health policies to address the growing threat of antibiotic resistance in *Klebsiella pneumoniae*.

### Future directions

The research on Cow102 and its mobile genetic elements, such as IncFIB(K), is being expanded to understand their role in the dissemination of antimicrobial resistance genes among different bacterial groups in raw milk. However, further investigation is also required to characterize the capsular K type of strain Cow102.

### Nucleotide sequence accession number

The draft genome sequence of *Klebsiella pneumoniae subsp. pneumoniae strain Cow102* has been deposited at DBJ/EMBL/GenBank under the accession JAZHQA000000000. The version described in this paper is version JAZHQA010000000.The raw sequence reads were deposited in DDBJ under BioProject number PRJNA1073760 and BioSample number SAMN39839089. Genome annotation, 16s rRNA, and bacteriophages of Cow102 strains reported in this study has been made available on Figshare: 10.6084/m9.figshare.25152032.

## Materials and methods

### Bacterial isolation and growth conditions

The current report stemmed from a 2019 study that sought to identify functional bacteria in animal milk [13, 14]. A synopsis of the sample collection and bacterial isolation conducted is provided here. Cow milk samples (*n* = 20) were collected from dairy farms in Sokoto State, Nigeria, between May and June 2019. Each sample was subjected to six dilution series. Bacteria were isolated on De-Mann Rogosa Sharpe (MRS) agar plates supplemented with D-Sorbitol and incubation of inoculated plates was performed in anaerobic conditions at 37 °C for 24 h.

### Antimicrobial susceptibility testing and screening of the ESBL production

Antimicrobial susceptibility testing (AST) was done using the agar diffusion method and the Clinical and Laboratory Standards Institute (CLSI) M100 guideline (31st edition) for drug selection and interpretation of result. Briefly, Mueller–Hinton agar plates (20 ml) were overlaid with MacFarland standardized broth containing the *Klebsiella pneumoniae* subsp. *pneumoniae* strain Cow102. The plates were then treated with a selection of antibiotics from various classes: aminoglycosides (streptomycin [10 µg]) quinolones (ciprofloxacin [5 µg], nalidixic acid [30 µg]) beta-lactams (amoxicillin + clavulanic acid [20/10 µg], ampicillin [10 µg], cefotaxime [30 µg], ceftazidime [30 µg], piperacillin [100 µg]), folic acid synthesis inhibitor (trimethoprim/sulfamethoxazole [1.25/23.75 µg]), monobactams (aztreonam [30 µg]) and other antibiotics (fosfomycin [200 µg], tetracycline [30 µg]). Plates were incubated at 37 °C for 24 h under aerobic conditions, thereafter the diameters of the inhibition zones were measured. The strain was considered susceptible or resistant, using the breakpoints established by CLSI.

ESBL-production was suspected due to reduced susceptibility to Ceftazidime (30 µg) and Cefotaxime (30 µg) in the AST. The Combination Disc Test (CDT), as recommended by the CLSI, was employed to confirm the production of ESBL. In summary, clavulanic acid (10 µg) was combined with each of cefotaxime (30 µg) and ceftazidime (30 µg). The test was considered positive when an increase in the growth-inhibitory zone around either the ceftazidime or the cefotaxime disk with clavulanic acid was ≥ 5 mm the diameter around the disk containing ceftazidime or cefotaxime alone.

### Haemolysis test

Haemolytic phenotype was tested on blood agar plates (Blood Agar Base number 2; Oxoid, Basingstoke, UK) containing defibrinated sheep erythrocytes 5% v/v. Production of haemolysis was read after overnight incubation at 37 °C.

### DNA extraction and sequencing

High-quality genomic DNA was extracted from pure pellets of *K. pneumoniae* subsp. *pneumoniae* Cow102 using the Quick-DNA fungal/bacterial miniprep kit (Zymo Research). Genomic library was prepared using Illumina TruSeq Nano DNA library preparation kit. Pair-end sequencing (2 × 150 base pairs) was performed using Illumina NovaSeq 6000 at Novogene Bioinformatics Technology Co. Ltd. South Africa. The quality of the library was ascertained on a Qubit 2.0 Fluorometer (Thermo Scientific).

### Genome assembly, annotation, and alignment

The quality of sequence reads was determined using FastQC Version 0.12.0 [49]. Adapter trimming, quality filtering, and per-read quality pruning was performed using fastp software [50]. The sequence reads were merged using PEAR v0.9.6 [51]. The filtered paired-end reads were *de novo* assembled using SPAdes v3.15.3 [52]. Genome quality and completeness was evaluated using CheckM v1.0.18 [53] while quality assessment of the assembled sequence was done using QUality ASsessment Tool (QUAST) v5.2.0 [54]. Genome annotation was performed using the RASTk version 1.073 [55].

### Taxonomic assignment, Multilocus sequence typing (MLST) and capsular typing

Taxonomic classification of the strain was done using kraken2 v2.1.3 [56] and the Genome Database Taxonomy (GTDB-Tk) v2.3.2 [57]. High quality genomes from The National Center for Biotechnology Information (NCBI) Reference sequence (RefSeq) database [1, 58] database [ were retrieved for the calculation of evolutionary distances. Reference genomes closely similar to the cow102 strain were identified by Mash/MinHash algorithm [59], protein families were identified using Protein Families for the Microbial Genomes database (PATtyFam) [60]. The protein sequences were aligned using MUSCLE v5 [61] and the nucleotides for each of those sequences mapped to the protein alignment. The resulting alignments were concatenated into a data matrix for phylogenetic analysis using RaxML v8.2.12 [62].

Multilocus sequence typing (MLST) of seven housekeeping genes (gapA, infB, mdh, pgi, phoE, rpoB and tonB) was performed by querying the Pasteur Institute (http://bigsdb.pasteur.fr/klebsiella/klebsiella.html) [63] using the MLST v2.220 software [64]. The Kaptive tool v2.0.4 [65] was used to determine capsular type (K-type and O-type).

### Identification of resistance determinants, virulence factors, phages and mobile genetic elements

The staramr tool v0.10 [66] was used to query the current genome against the ResFinder database v4.4.2 [67] for profiling of AMR genes and drug classes, the plasmidfinder database [68] for identification of plasmids. Using ABRicate v1.0.1 [69], virulence determinants were investigated by aligning the reads to the Virulence Factors Database (VFDB) [70]. ISEScan tool V1.7.2.3 [71] was used to identify IS elements on the ISFinder database [72]. Insertion sequence elements shorter than 400 base pairs or single copy IS elements without perfect terminal inverted repeats were not considered. Phages were identified using the PHASTER (PHAge Search Tool Enhanced Release) web tool (https://phaster.ca/) [73]. Default parameters were used for all tools except otherwise stated.

### Comparative genome

Genomic variation in Cow102 was determined by comparative genome analysis with nine (ASM990733v1, ASM990770v1, ASM990777v1, ASM990722v1, ASM990712v1, ASM2554830v1, ASM990709v1, ASM2554845v1, ASM2554839v1) *K. pneumoniae* strains of different sequence types isolated in Nigeria, all strains were obtained from the NCBI database. Detailed description of selected strains is presented in Supplementary data S3. Sequence alignment, pairwise average nucleotide identity (ANI) and sequence alignment percentages was performed using the CLC genomic workbench software v24.0. Thereafter, genome-wide variants were identified using MUMmer4’s DNAdiff tool v4.0 [74] using the *K. pneumoniae* subsp. *pneumoniae* cow102 assembly against each NCBI assembly. Structural relocations, translocations, and inversions were identified alongside total and aligned bases. Prior to running the DNAdiff tool, each assembly was filtered to remove contigs of < 1 kb in length to prevent short sequences from exaggerating structural variations between assemblies. In addition, Orthologous gene clusters were identified using OrthoFinder v2.5.5 [75]. The proteins of Cow102 was also compared to each genome obtained from GenBank, using the online platform OrthoVenn [76] with default parameters.

### Quality assurance

The genomic DNA used for sequencing was isolated from a single colony of the bacteria. The 16 S rDNA gene was extracted from the genome using extractseq version 5.0.0 [77][. The assessment of potential contamination of the genomic library by allochthonous microorganisms was achieved through BLAST annotation against NCBI microbial 16 S database. The extracted 16 S sequences have been made available on Figshare [21].

## Electronic supplementary material

Below is the link to the electronic supplementary material.

Supplementary Material 1

Supplementary Material 2

Supplementary Material 3

Supplementary Material 4

Supplementary Material 5

Supplementary Material 6

Supplementary Material 7

## Data Availability

The draft genome sequence of Klebsiella pneumoniae subsp. pneumoniae strain Cow102 has been deposited at DBJ/EMBL/GenBank under the accession JAZHQA000000000. The version described in this paper is version JAZHQA010000000.The raw sequence reads were deposited in DDBJ under BioProject number PRJNA1073760 and BioSample number SAMN39839089. Genome annotation, 16s rRNA, and bacteriophages of Cow102 strains reported in this study has been made available on Figshare: https://doi.org/10.6084/m9.figshare.25152032.
