# Supplementary material for: Genomic characterisation of an extended-spectrum β-Lactamase-producing Klebsiella pneumoniae isolate assigned to a novel sequence type (6914)
Source: Gut Pathog. 2024 Nov 15;16:69. doi: 10.1186/s13099-024-00662-4 (PMC11566244; doi:10.1186/s13099-024-00662-4)
Supplement: Supplementary file 1 — Supplementary Material 1 [file 13099_2024_662_MOESM1_ESM.docx]

Supplementary table 1: Virulence genes of *Klebsiella pneumoniae* subsp. *pneumoniae* strain Cow102.

| Virulence factor | Related Gene | Function |
| --- | --- | --- |
| Adherence | *fimC* | Periplasmic chaperone |
| Adherence | *fimD* | Outer membrane usher protein |
| Adherence | *fimH* | Type 1 fimbrial adhesin precursor |
| Adherence | *fimK* | Transcriptional regulator |
| Adherence | *fleQ* | Transcriptional regulator |
| Adherence | *htpB* | Hsp60, heat shock protein |
| Adherence | *pilB* | Type IV-A pilus assembly |
| Adherence | *pilR* | Two-component response regulator |
| Adherence | *pilT* | Twitching motility protein |
| Adherence | *rpoN* | RNA polymerase factor sigma-54 |
| Adherence | *rpoS* | RNA polymerase sigma factor |
| Adherence | *tapT* | Twitching |
| Adherence | *tufA* | Elongation factor |
| Adherence | *vfr* | Camp-regulatory protein |
| Adherence | *yagX/ecpC* | E. coli common pilus usher |
| Antimicrobial activity/ Competitive advantage | *mtrD* | Multiple transferable resistance system protein |
| Antimicrobial activity/ Competitive advantage | *acrA* | Acriflavine resistance protein |
| Antimicrobial activity/ Competitive advantage | *acrB* | Acriflavine resistance protein |
| Biofilm | *algW* | Algw protein |
| Biofilm | *luxS* | S-ribosylhomocysteinase |
| Biofilm | *mrkB* | Fimbrial chaperone protein |
| Biofilm | *mrkC* | Fimbrial biogenesis outer membrane usher protein |
| Biofilm | *mrkD* | Fimbrial adhesin protein precursor |
| Biofilm | *mrkF* | Type 3 fimbrial minor pilin subunit |
| Biofilm | *mrkH* | Transcriptional activator |
| Biofilm | *mrkJ* | Phosphodiesterase |
| Biofilm | *mucD* | Serine protease mucD precursor |
| Biofilm | *pgaA* | Poly-beta-1,6 N-acetyl-D-glucosamine export porin |
| Effector delivery system | *clpB* | Type VI secretion system |
| Effector delivery system | *clpV/tssH* | Type VI secretion system |
| Effector delivery system | *clpV1* | Type VI secretion system AAA+ family |
| Effector delivery system | *exeD* | General secretion pathway protein |
| Effector delivery system | *exeE* | General secretion pathway protein |
| Effector delivery system | *exeF* | General secretion pathway protein |
| Effector delivery system | *exeG* | General secretion pathway protein |
| Effector delivery system | *exlA* | Hemolysin |
| Effector delivery system | *gspE* | General secretion pathway protein |
| Effector delivery system | *gspG* | General secretion pathway protein |
| Effector delivery system | *icmF/tssM* | Type VI secretion protein |
| Effector delivery system | *impA/tssA* | Type VI secretion system protein |
| Effector delivery system | *PA1663* | Transcriptional regulator |
| Effector delivery system | *PA2359* | Transcriptional regulator |
| Effector delivery system | *tssF* | Type VI secretion system baseplate subunit |
| Effector delivery system | *tssG* | Type VI secretion system baseplate subunit |
| Effector delivery system | *tssH* | Type VI secretion system |
| Effector delivery system | *tssH-5/clpV* | Clp-type chaperone protein |
| Effector delivery system | *tssM* | Type VI secretion system membrane subunit |
| Effector delivery system | *vasE/tssK* | Type VI secretion system baseplate subunit |
| Effector delivery system | *vasH* | Sigma-54 dependent transcriptional regulator |
| Effector delivery system | *vgrG/tssI* | Type VI secretion system tip protein |
| Effector delivery system | *vipB/tssC* | Type VI secretion system contractile sheath large subunit |
| Effector delivery system | *xcpR* | General secretion pathway protein |
| Effector delivery system | *yst1E* | Type II secretion system |
| Exotoxin | *cyaB* | Cyclolysin secretion ATP-binding protein |
| Exotoxin | *rtxB* | RTX toxin transporter |
| Immune modulation | *galF* | Galu regulator |
| Immune modulation | *gmhA/lpcA* | Phosphoheptose isomerase |
| Immune modulation | *KP1_RS17220* | Glycosyltransferase |
| Immune modulation | *KP1_RS17230* | Glycosyltransferase |
| Immune modulation | *KP1_RS17240* | DUF4422 domain-containing protein |
| Immune modulation | *KP1_RS17345* | Capsule assembly Wzi family protein |
| Immune modulation | *rfaD* | ADP-L-glycero-D-mannoheptose-6-epimerase |
| Immune modulation | *rfaJ* | Alpha-1,6 Glc transferase |
| Immune modulation | *rfbA* | O-antigen export ABC transporter permease |
| Immune modulation | *rfbB* | O-antigen export ABC transporter ATP-binding protein |
| Immune modulation | *rfbD* | UDP-galactopyranose mutase |
| Immune modulation | *rfbK1* | O9 family phosphomannomutase |
| Immune modulation | *rffG* | Dtdp-glucose 46-dehydratase |
| Immune modulation | *waaA* | Lipopolysaccharide core biosynthesis protein |
| Immune modulation | *waaC* | 3-deoxy-D-manno-octulosonic-acid (KDO) transferase |
| Immune modulation | *waaF* | Heptosyltransferase I |
| Invasion | *ibeB* | Cu(+)/Ag(+) efflux RND transporter outer membrane channel |
| Invasion | *ompA* | Outer membrane protein |
| Motility | *flrA* | Sigma-54 dependent transcriptional activator |
| Motility | *flmH* | Short chain dehydrogenase/reductase family oxidoreductase |
| Motility | *nueA* | Neua protein |
| Nutritional/Metabolic factor | *entA* | 2,3-dihydroxybenzoate-2,3-dehydrogenase |
| Nutritional/Metabolic factor | *entC* | Isochorismate synthase |
| Nutritional/Metabolic factor | *entE* | Enterobactin synthase subunit |
| Nutritional/Metabolic factor | *entF* | Enterobactin synthase subunit |
| Nutritional/Metabolic factor | *entS* | Enterobactin exporter |
| Nutritional/Metabolic factor | *fbpC* | Iron(III) ABC transporter, ATP-binding protein |
| Nutritional/Metabolic factor | *fepA* | Ferrienterobactin outer membrane transporter |
| Nutritional/Metabolic factor | *fepD* | Iron-enterobactin transporter membrane protein |
| Nutritional/Metabolic factor | *fes* | Enterobactin/ferric enterobactin esterase |
| Nutritional/Metabolic factor | *iroE* | Siderophore esterase |
| Nutritional/Metabolic factor | *iroN* | Salmochelin receptor |
| Nutritional/Metabolic factor | *iutA* | Ferric aerobactin receptor |
| Nutritional/Metabolic factor | *mgtB* | Mg2+ transport protein |
| Nutritional/Metabolic factor | *pchI* | ABC transporter ATP-binding protein |
| Others | *icl* | Isocitrate lyase |
| Regulation | *cdpA* | Cyclic di-GMP phosphodiesterase |
| Regulation | *phoP* | Response regulator in two-component regulatory system with PhoQ |
| Regulation | *phoQ* | Sensor protein |
| Regulation | *sigA/rpoV* | RNA polymerase sigma factor |
| Regulation | *pmrA* | Response regulator |
| Regulation | *pmrB* | Sensory kinase |
| Regulation | *relA* | Probable GTP pyrophosphokinase |
