# Supplementary figures and images for: Genomic characterisation of an extended-spectrum β-Lactamase-producing Klebsiella pneumoniae isolate assigned to a novel sequence type (6914)
Source: Gut Pathog. 2024 Nov 15;16:69. doi: 10.1186/s13099-024-00662-4 (PMC11566244; doi:10.1186/s13099-024-00662-4)

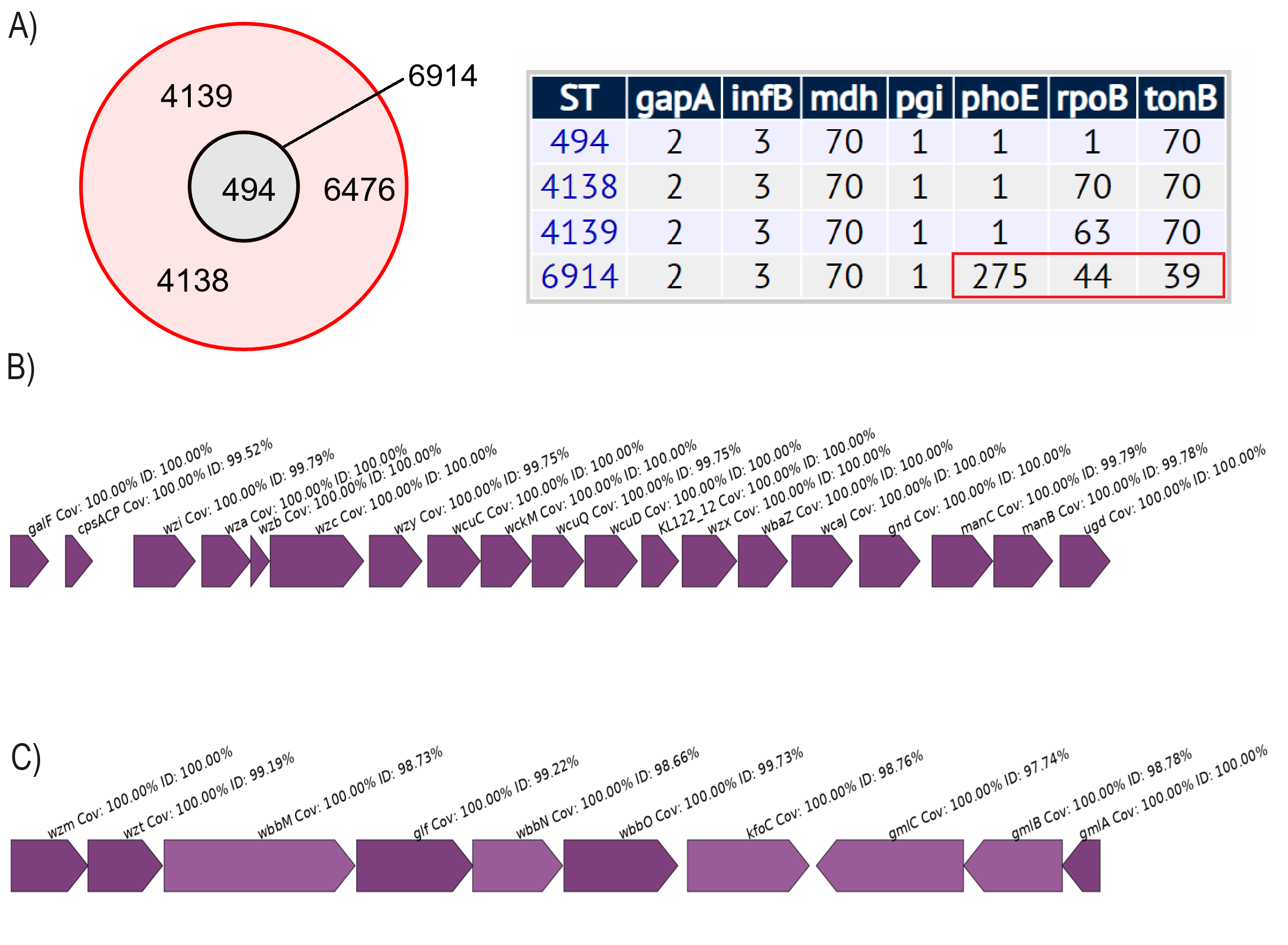

Supplement: Supplementary file 2 — Supplementary Material 2 [file 13099_2024_662_MOESM2_ESM.tif]

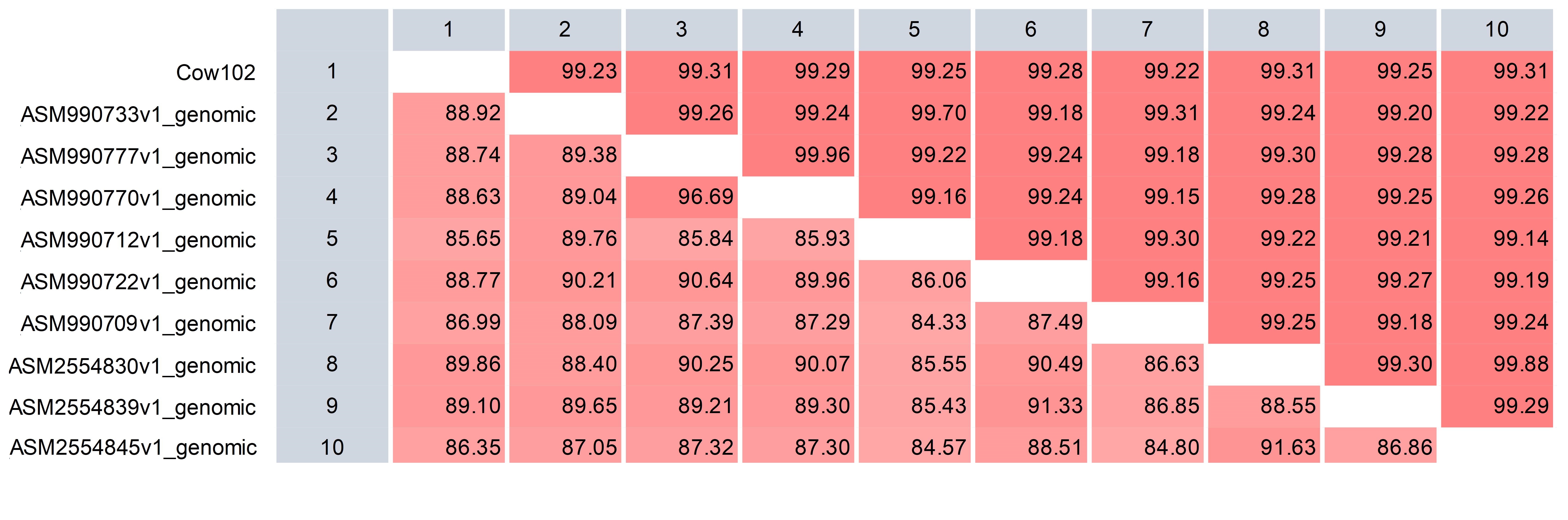

Supplement: Supplementary file 3 — Supplementary Material 3 [file 13099_2024_662_MOESM3_ESM.jpg]
